# Cross-sectional study of cognitive impairment and visual impairment among the elderly population in residential care in India: the Hyderabad Ocular Morbidity in Elderly Study (HOMES)

**DOI:** 10.1136/bmjopen-2024-084348

**Published:** 2024-07-22

**Authors:** Srinivas Marmamula, Thirupathi Reddy Kumbham, Joshua R Ehrlich, Suvarna Alladi, David E Bloom, David S Friedman

**Affiliations:** 1 Allen Foster Community Eye Health Research Centre, Gullapalli Pratibha Rao International Centre for Advancement of Rural Eye care, L V Prasad Eye Institute, Hyderabad, India; 2 Brien Holden Institute of Optometry and Vision Sciences, Prof. Brien Holden Eye Research Centre, L V Prasad Eye Institute, Hyderabad, India; 3 Department of Biotechnology / Wellcome Trust India Alliance, L V Prasad Eye Institute, Hyderabad, India; 4 Department of Global Health and Population, Harvard T.H. Chan School of Public Health, Boston, MA, USA; 5 Department of Ophthalmology & Visual Sciences, University of Michigan, Ann Arbor, MI, USA; 6 Survey Research Centre, Institute for Social Research, University of Michigan, Ann Arbor, MI, USA; 7 National Institute of Mental Health and Neurosciences, Bengaluru, India; 8 Department of Ophthalmology, Massachusetts Eye and Ear, Harvard Medical School, Boston, MA, USA

**Keywords:** epidemiologic studies, geriatric medicine, public health

## Abstract

**Abstract:**

**Objective:**

To report the relationship between visual impairment (VI) and cognitive impairment (CI) among the older population living in residential care homes in Hyderabad, India.

**Study design:**

Cross-sectional study.

**Setting:**

41 homes for the aged centres in the Hyderabad region.

**Participants:**

965 participants aged ≥60 years from homes for the aged centres.

**Primary outcome measures:**

Visual impairment and cognitive impairment.

**Methods:**

The Hindi mini-Mental Status Examination (HMSE) questionnaire was used to assess the cognitive function. The final HMSE score was calculated after excluding vision-dependent tasks (HMSE-VI). A detailed eye examination was conducted, including visual acuity (VA) measurement for distance and near vision, using a standard logarithm of the minimum angle of resolution chart under good illumination. CI was defined as having a HMSE-VI score of ≤17. VI was defined as presenting VA worse than 6/12 in the better-seeing eye. Near VI (NVI) was defined as binocular presenting near vision worse than N8 and distance VA of 6/18 or better in the better-seeing eye. Multiple logistic regression was done to assess the association between VI and CI.

**Results:**

The mean age (±SD) was 74.3 (±8.3) years (range: 60–97 years). There were 612 (63.4%) women, and 593 (61.5%) had a school education. In total, 260 (26.9%; 95% confidence intervals: 24.2 to 29.9) participants had CI. The prevalence of CI among those with VI was 40.5% compared with 14.6% among those without VI (p<0.01). The logistic regression analysis showed that the participants with VI for distance vision had three times higher odds of having CI (OR 3.09; 95% confidence intervals: 2.13 to 4.47; p<0.01). Similarly, participants with NVI had two times higher odds of having CI (OR 2.11; 95% confidence intervals: 1.36 to 3.29; p<0.01) after adjusting for other covariates.

**Conclusions:**

CI was highly prevalent among those with distance and near VI. VI was independently and positively associated with CI after adjusting for potential confounders. Interventions can be planned to address VI in this vulnerable population which could have a ripple effect in preventing cognitive decline.

**STRENGTHS AND LIMITATIONS OF THIS STUDY**

- Large sample size, comprehensive eye examination including visual acuity assessment and use of a standard questionnaire to assess cognition.
- Vision dependent tasks in the cognition assessment were excluded to control for the effect of visual impairment on cognition test results.
- Prevalence of cognitive impairment among those with distance visual impairment and those with near vision impairment with normal vision for distance are reported.
- Participants with severe grades of hearing impairment could not complete the cognition assessment, and hence, the overall prevalence of cognitive impairment could have been underestimated.
- The results from this study cannot be extrapolated to community-dwelling older people, as it was carried out among older people living in residential care in an urban area.

## Introduction

 Visual impairment (VI) and cognitive impairment (CI) are twin public health challenges that predominantly affect older people. VI affects over 1.1 billion people worldwide, with a higher prevalence in older age groups.^1^ By the year 2050, 1.7 billion are projected to be affected.^1^ VI significantly impacts the emotional, social and economic well-being of an individual. It adversely affects the individual’s quality of life and leads to a substantial loss in economic productivity.^2 3^ VI also increases the risk of mortality.^4^ In India, one out of every three adults aged 60 years and older has VI.^5 6^ However, approximately 9/10 cases of VI in India can be addressed using cost-effective interventions, such as spectacles and cataract surgery.^7^

Dementia is a severe form of CI that affected over 57.4 million people worldwide in 2019. This number is expected to rise to approximately 152 million (117% increase) by 2050.^8^ Moreover, this increase is expected to affect low-income and middle-income countries the most.^8^ The prevalence of CI in population-based studies ranges from 3%–44%.^9^,^11^ The Longitudinal Ageing Study in India-Diagnostic Assessment of Dementia (LASI-DAD), one of the largest nationally representative studies conducted in India, reported the prevalence of dementia as 7.4% (affecting 8.8 million people).^12^

Vision and cognition seem to be intricately interwoven, as many cross-sectional and longitudinal studies have revealed a positive association between them.^13^,^16^ In a recent systematic review of 110 studies, 91 reported a significant positive association between VI and CI.^17^ However, this systematic review included only a few studies from developing countries and none from India.^17^ In India, the first study investigating the relationship between cognition and vision reported a positive association between VI and CI.^18^ It also found that cognitive function was lower with higher levels of VI, even when vision-dependent cognitive tests were excluded.^18^

Understanding the relationship between VI and CI is important to plan interventions. VI could be a modifiable risk factor for CI and dementia. Theoretically, visual stimulation affects the cognitive load on the brain and prevents cognitive decline by improving physical and social functioning and overall quality of life.^19^ A few longitudinal studies have indicated improvements in cognitive functions after cataract surgery.^20 21^ Both CI and VI are more common among older people. India is ageing, and projections are that by 2050, every fifth Indian will be aged 60 years or older, accounting for 320 million people. In addition to this demographic transition, many social changes are evident, such as new living arrangements and family structures due to urbanisation, including residential homes for the aged.^22^ At this crucial juncture, understanding the impact of these changes on the lives of the elderly is vital.

In India, homes for the aged are a recent and emerging concept.^23^ These homes are diverse in terms of scope, services provided and the number of individuals residing in them.^23^ There are no standardised guidelines for admission into these homes. While most homes admit people aged 60 and older, few even admit those aged 50 years and older. Few homes restrict admissions to older people with independent mobility while others also include those who need assistance for mobility and a few who are bedridden. In addition, there are specific homes which are typical nursing homes or rehabilitation centres for the older people with disabilities and those who are bedridden. Non-governmental, religious or voluntary organisations with support from the government and philanthropists manage these homes. In some homes, the elderly themselves or their kin pay the monthly or annual ‘user fee’.^23^

With this background, the Hyderabad Ocular Morbidity in Elderly Study (HOMES) was designed to assess the prevalence, causes and risk factors of VI among elderly individuals living in homes for the aged in Hyderabad, India.^23^ A secondary objective of this study was to assess the relationship between cognitive function and VI. The prevalence of VI, its causes and risk factors have been reported previously.^24^ The current study focuses on cognitive function and VI among older adults living in residential homes for the aged in Hyderabad, India.

## Methods

### Study participants

The HOMES included 1515 participants enumerated from 41 residential care homes for the aged in Hyderabad.^23 24^ Residents aged ≥60 years residing in these homes for at least 1 month at the time of enumeration and who agreed to participate were included in the study. Of the 1182 participants examined in HOMES, 217 were bedridden or had serious medical issues and were excluded from these analyses. Therefore, data from the remaining 965 participants were included in the analyses.

### Assessment of cognitive function

The Hindi mini-Mental Status Examination (HMSE) questionnaire is used to assess cognition in India.^25^ Similar to the Mini-Mental Status Examination (MMSE)-blind, the final HMSE score was calculated after excluding vision-dependent tasks (HMSE-VI).^1526^,^28^ The maximum possible score was 22 points compared with the 30 in the conventional HMSE.^25^ A high HMSE-VI score was defined as >17, and a low HMSE-VI score was defined as ≤17. Other researchers have used a similar cut-off for the MMSE-blind scale.^15^ In this study, trained investigators conducted the HMSE-VI assessments in the local language in homes for the aged facilities.

### Eye health assessment

A detailed eye examination was performed after the interview, as reported in our previous publications.^23 24 29^ This assessment included visual acuity measurement for distance and near vision using a standard logarithm of the minimum angle of resolution (logMAR) chart under good illumination. Visual acuity was assessed with spectacles, if available. Distance visual acuity was assessed at 3 m in a well-illuminated room (at least 180 lux), and near vision was assessed at a fixed distance of 40 cm. Visual acuity charts with tumbling E optotypes and English letter alphabets were used for assessment. An anterior segment examination using a portable handheld slit lamp and fundus imaging were carried out. Interviews and clinical eye examinations were done on different days to ensure that the participants had adequate rest between the assessments. The following two definitions were used for distance VI based on the presenting visual acuity in the better eye:

1. Definition 1: a visual acuity threshold worse than 6/12 (mild VI or worse).
2. Definition 2: a visual acuity threshold worse than 6/18 (moderate VI or worse).

Both definitions are in line with recent definitions of VI.^24^ Near VI (NVI) was defined as presenting binocular near vision worse than N8 among those with no VI for distance vision (presenting visual acuity of 6/18 or better in the better-seeing eye). N8 corresponds to the font size commonly used in the newsprint.

### Other covariates

Trained field investigators collected data on personal and demographic information, such as age, gender and highest education level, in face-to-face interviews. The following covariates were included : age group (60–69, 70–79 and ≥80 years); gender (male or female); education: higher education (graduation and above), primary or secondary school only (1–12 years of education) or no formal education; the type of home: private (where the individual or their kin pay a monthly or annual user fee), subsidised homes (where the individual or their kin pay only a part of the fee and the remainder is supported from other funding sources), and free homes (where the individual does not pay and homes are supported by external funding) and self-report of diabetes and hypertension (present/absent).

### Patient and public involvement

Patients and other members of the public were not involved in the study.

### Data analysis

Statistical analyses were conducted using Stata V.14 (Stata Corp, College Station, Texas, USA). The prevalence of CI was calculated and presented with 95% confidence intervals. A Pearson χ^2^ test was used to compare categorical variables, and the Student’s t-test and ANOVA (Analysis of Variance) were used for continuous variables. In multiple logistic regression analysis, VI was used as an outcome variable, and its association with CI was assessed with the sociodemographic variables (age, gender and level of education) as covariates. The adjusted OR (odds ratio) with 95% confidence intervals have been presented. For all analyses, the statistical significance was set at p<0.05 (two-tailed); however, the exact p-values have been reported.

## Results

### Characteristics of the participants

The mean age (±SD) of participants was 74.3 (±8.3) years (range: 60–97 years). There were 612 (63.4%) female participants, and 593 (61.5%) had attended at least some primary school. In terms of the type of home, 382 (39.6%), 425 (44.0%) and 158 (16.4%) were living in private, subsidised and free homes, respectively. Diabetes and hypertension were reported by 281 (29.12%) and 559 (59.7%) participants, respectively. In total, 459 (47.6) participants had VI using definition 1 (<6/18); 243 (25.2%) participants had VI using definition 2 (<6/12) and 355/722 (49.2%) participants had NVI (table 1).

The overall prevalence of CI was 26.9% (95% confidence intervals: 24.2 to 29.9; n=260). In a total of 459/965 (47.6%) participants who had VI for distance using the definition 1 (<6/12), 186 (40.5%; 95% confidence intervals: 36.0 to 45.1) participants had CI. Similarly, 243/965 (25.2%) participants who had VI using definition 2 (<6/18), 122 (50.2%; 95% confidence intervals: 43.7 to 56.7) participants had CI. Of 355/722 (49.2%) participants who had NVI, 92 (25.9%; 95% confidence intervals: 21.4 to 30.8) participants had CI.

**Table 1:** Characteristics of the study participants

Mean (SD) age in years	74.3 (8.4)	
**Hindimini-Mental Status Examination-VisualImpairment(HMSE-VI) scores—mean (SD)**	**18.1(3.9)**	
	n	%
Age group (years)		
60–69	284	29.4
70–79	387	40.1
80 and above	294	30.5
Gender		
Male	353	36.6
Female	612	63.4
Education level		
Higher education	196	20.3
School education	593	61.5
No education	176	18.2
Type of home		
Paid home	382	39.6
Subsidised home	425	44.0
Free home	158	16.4
Diabetes		
No	684	70.9
Yes	281	29.1
Hypertension		
No	406	42.1
Yes	559	57.9
Visual impairment (<6/12)		
No	506	52.4
Yes	459	47.6
Visual impairment (<6/18)		
No	722	74.8
Yes	243	25.2
Near visual impairment (n=722)		
No	367	50.8
Yes	355	49.2

### Association between VI and CI

The logistic regression analysis showed that the participants with VI for distance vision had higher odds for CI using both definitions (<6/12 and 6/18). Participants with VI had four times higher odds of having CI compared with those without VI. This association remained significantly high after adjusting for age, gender and education in model 1. The association remained significantly high after controlling for other covariates, including type of home, diabetes and hypertension, in model 2. Similarly, the participants with NVI had two times higher odds to have CI compared with those without NVI. This association persisted after adjusting for other covariates in models 1 and 2 (table 2).

**Table 2:** The effects of personal and demographic characteristics on cognitive impairment (multiple logistic regression analysis)

	Distance visual Impairment (<6/12)		Distance visual impairment (<6/18)		Near visual impairment (worse than N8)	
	Odds Ratio (95% Confidence intervals)	P value	Odds Ratio (95% Confidence intervals)	P value	Odds Ratio (95% Confidence intervals)	P value
**Model 1—CrudeOddsRatio**						
Cognitive impairment						
No	Reference		Reference		Reference	
Yes	3.98 (2.92 to 5.42)	<0.01	4.27 (3.12 to 5.83)	<0.01	2.44 (1.65 to 3.60)	<0.01
**Model 2—adjusted for age, genderand education**						
Cognitive impairment						
No	Reference		Reference		Reference	
Yes	3.09 (2.13 to 4.47)	<0.01	3.04 (2.13 to 4.35)	<0.01	2.12 (1.36 to 3.29)	<0.01
**Model3—adjusted for age, gender and education, type of home, diabetesand hypertension**						
Cognitive impairment						
No	Reference		Reference		Reference	
Yes	3.16 (2.19 to 4.56)	<0.01	2.91 (2.00 to 4.25)	<0.01	2.13 (1.36 to 3.30)	<0.01

### Categories of VI and CI

The prevalence of CI varied from 14.6% among those with no VI to 60% among those with blindness. The mean HMSE-VI scores were lower among those with severe VI (one-way ANOVA; p<0.01) (table 3). The mean HMSE-VI scores were lower for those with VI caused due to cataract compared with other causes of VI (table 4).

**Table 3:** Prevalence of cognitive impairment and mean (±SE of the mean) Hindi mini-Mental Status Examination-Visual Impairment (HMSE-VI) scores with categories of visual impairment (VI)

	Number in the sample	Prevalence of cognitive impairmentn (%)	Mean HMSE-VI score (±SE of the mean)
Visual impairment (VI) category			
No VI	506	74 (14.6)	19.5 (0.1)
Mild VI	216	64 (29.6)	17.5 (0.3)
Moderate VI	204	100 (49.0)	15.8 (0.3)
Severe VI	24	13 (54.2)	15.7 (1.0)
Blindness	15	9 (60.0)	14.7 (1.2)
Total	965	260 (26.9)	18.1 (0.1)
VI—6/12 Definition			
No VI	506	74 (14.6)	19.5 (0.1)
VI	459	186 (40.5)	16.6 (0.2)
VI—6/18 Definition			
No VI	722	138 (19.1)	18.9 (0.1)
VI	243	122 (50.2)	15.8 (0.1)

**Table 4:** Mean (±SE of the mean) of Hindi mini-Mental Status Examination-Visual Impairment (HMSE-VI) scores with causes of visual impairment.

Causes	Number (%)	Mean HMSE-VI score (±SE of the mean)
No visual impairment	722 (74.8)	18.9 (0.1)
Uncorrected refractive errors	76 (7.9)	16.2 (0.5)
Cataract	112 (11.6)	15.3 (0.4)
Other causes	55 (5.7)	16.1 (0.6)
Total	965 (100)	18.1 (0.1)

## Discussion

CI was significantly associated with VI after adjusting for potential demographic and socioeconomic confounders. A dose-response relationship was observed, with a higher prevalence of CI in those with worse grades of VI. Half of the participants with VI, using the 6/18 definition, had CI. Though MMSE and HMSE are the most common tools to assess cognitive function, most studies have not excluded vision-dependent tasks.^17^ A recent study that included a sample of participants from the Longitudinal Ageing Study in India presented its results after excluding vision-dependent tasks from a comprehensive cognitive test battery.^18^ This study noted poorer cognitive function among those with VI, which is consistent with our findings.^18^ Though there is a positive association of VI with the level of education and age, the association between VI and CI remained independent of these risk factors. NVI was also associated with CI after adjusting for other covariates. Similar findings are reported from a study conducted in the older populations in Israel.^30^

Several hypotheses could explain this strong and consistent association between CI and VI.^19 31 32^ One hypothesis is attributed to the common pathway that affects the ageing brain and visual system, as shown in a few studies that investigated vision and cognition.^31 32^ It is hypothesised that VI hinders social interactions, reduces physical activities and adversely affects the performance of vision-dependent tasks, resulting in cognitive decline and dementia.^17^ Participation in cognitively stimulating activities and physical activity is shown to delay the onset of dementia.^33 34^

One out of every four elderly individuals living in homes for the aged in Hyderabad, India had CI. These findings are consistent with other studies that have reported the prevalence of CI among older adults in India, ranging from <5% to 44%.^9^,^1135^ However, those studies were conducted in community settings, as opposed to the homes for the aged included in this study. One study from Kerala that included a smaller subsample of elderly residents in homes for the aged centres reported a prevalence of 32.4% in paid homes and 42.7% in free homes compared with 21.9% in the community-dwelling elderly population.^39^ Previous studies that investigated the prevalence of CI in India did not exclude participants with sensory impairments, which might have impacted the results and prevalence of CI.

The prevalence of CI increased with age in our study. Evidence from previous studies on the association between CI and older age, both from India and other countries, is equivocal.^17^ The evidence also points to a positive association between gender and CI, both from India and other countries.^40 41^ Women consistently have a higher prevalence of CI than men across studies.^40 41^ In this study, the prevalence of CI among women was almost twice that of men. A higher prevalence of CI was observed among those with lower levels of education, which is consistent with the results from other studies conducted in India.^9^,^11^ The education-cognition relationship is complex. Individuals with higher levels of education are likely to perform better on the cognitive test than those without any formal education. Moreover, individuals with higher levels of education are more likely to have more social interactions, cognitively active work lives and better socioeconomic status, slowing down the process of cognitive decline compared with their age-matched peers.^28 42^

The United Nations Decade of Healthy Ageing (2021–2023) provides the framework for the integrated care of older adults, including long-term care.^43 44^ The research on late-life health and well-being is a cornerstone for achieving the goal of healthy and active ageing. The key is to identify the modifiable risk factors and design interventions to address them holistically. In line with this initiative, the current research investigated the relationship between two key public challenges that affect older adults in residential care to provide valuable insights into the provision of services. This study also highlights the need to screen for other dimensions of health and wellbeing such as cognition among older people seeking eye care and make appropriate referrals for services where needed.

Though several studies have investigated CI, only a few have described the relationship between CI and VI in India, and none has reported the association between CI and VI among the older population in residential care settings. There are no reports on NVI and CI in India. This study included a large sample of individuals from homes for the aged and an in-depth vision and eye health assessment, including the causes of VI. Unlike the previous studies done in India, we excluded the vision-dependent tasks from the HMSE to assess cognitive function independent of the severity of VI to provide a more realistic prevalence of CI. That said, the impact of VI on cognitive tests is not reported from India. Researchers from Singapore presented a vision-independent battery to assess cognitive function among the older people with VI.^45^ To our knowledge, no publication has reported the HMSE-VI scores among the visually impaired population in India before this study.

This study had a few limitations. Participants with severe hearing loss did not complete the HMSE questionnaire, as it was difficult to communicate and elicit reliable responses. Moreover, those with mobility challenges or who were bedridden were not included. Therefore, we may have underestimated the prevalence of poor cognitive function and its association with VI. In addition to severity, the duration of VI could also impact cognitive function. However, this study could not assess the duration of vision loss. Given the cross-sectional design of the study, a causal relationship between VI and CI could not be established. Finally, the study included only individuals from homes for the aged in an urban region of India. Therefore, our results cannot be generalised to the population at large.

In conclusion, VI is associated with CI in older adults in residential care in Hyderabad, Telangana, India. As a large proportion of VI is avoidable, interventions to address VI might cause a positive ripple effect on cognitive functions and improve the well-being of older people in residential care, though additional interventional research is warranted.

## Data Availability

Data are available upon reasonable request.
