# Supplementary material for: Cross-sectional study of cognitive impairment and visual impairment among the elderly population in residential care in India: the Hyderabad Ocular Morbidity in Elderly Study (HOMES)
Source: BMJ Open. 2024 Jul 22;14(7):e084348. doi: 10.1136/bmjopen-2024-084348 (PMC11407191; doi:10.1136/bmjopen-2024-084348)
Supplement: Supplementary file 1 [file bmjopen-2024-084348.reviewer_comments.pdf]

PEER REVIEW HISTORY

BMJ Open publishes all reviews undertaken for accepted manuscripts. Reviewers are asked to complete a checklist review form (**http://bmjopen.bmj.com/site/about/resources/checklist.pdf**) and are provided with free text boxes to elaborate on their assessment. These free text comments are reproduced below.

ARTICLE DETAILS

**TITLE (PROVISIONAL)**	A cross-sectional study cognitive impairment and visual impairment among the elderly population in residential care in India: The Hyderabad Ocular Morbidity in Elderly Study (HOMES)
**AUTHORS**	Marmamula, Srinivas; Kumbham, Thirupathi Reddy; Ehrlich, Joshua; Alladi, Suvarna; Bloom, David; Friedman, David

VERSION 1 – REVIEW

**REVIEWER**	Drewes, Yvonne<br>Leiden University Medical Center, Public Health and Primary Care
**REVIEW RETURNED**	20-Feb-2024

**GENERAL COMMENTS**	Interesting manuscript, well written and very well structured.<br><br>Only some smaller points:<br>Regarding the abstract: The last sentence, 'Interventions to address VI might be a viable approach to improve cognitive function and the quality of life and well-being of elderly residents in residential care' is too far from the results, as this is a crossectional design; causal conclusions cannot be formulated based on these results<br><br>Regarding the methods: were participants allowed to wear their spectacles during the vision test?<br><br>Regarding the limitation section: I was wondering what are the indications to be admitted to a nursing home in India? Is a combination of impairments are needed? This could strongly influence the findings of this specific population. It is already mentioned that the results cannot be generalized to the population at large, but the authors can be more explicit about the entry requirements for a nursing home and the way this influenced the outcomes of this study.

**REVIEWER**	Lee, Sumi<br>Howon University, Department of Nursing
**REVIEW RETURNED**	29-Feb-2024

**GENERAL COMMENTS**	Dear Srinivas Marmamula<br><br>I hope this message finds you well. I want to express our gratitude for submitting your manuscript entitled "Cognitive impairment and vision impairment among the elderly population in residential care in India: The Hyderabad Ocular Morbidity in Elderly Study (HOMES)]" to the BMJ reviewer. We appreciate the effort and dedication you have invested in your research.

	After careful consideration by our reviewer team, it is with regret that we are unable to accept your manuscript for publication at this time. Our decision is based on a comprehensive evaluation, and we would like to provide you with specific feedback to help you enhance the quality of your work for future submissions.<br><br>Abstract<br>In one sentence, unlike the other sentences, the p value is missing and needs to be corrected.<br>The logistic regression analysis showed that the participants with VI for distance vision were three times more likely to have CI (OR: 3.09; 95% Confidence Intervals: 2.13 – 4.47;<0.01).<br><br>Methods<br>I hope that the explanation of Near VI in Eye Health Assessment will be revised to be more concise and clear. In particular, it is difficult to understand what N8 means.<br><br>References<br>47 references are presented in the text. Among the references, it is recommended that numbers 2, 5, 34, 35, 36, and 41 be replaced with the most recent literature or deleted if possible.<br><br>We encourage you to address these issues in a revised version of your manuscript before resubmitting it for consideration.<br><br>We appreciate your understanding and hope that this feedback proves valuable in refining your work. If you have any questions or need further clarification, please feel free to contact us.<br><br>Thank you for considering BMJ Open as a venue for your research, and we wish you success in your future scholarly endeavors.<br><br>Sincerely,

VERSION 1 – AUTHOR RESPONSE

Reviewer: 1
Dr. Yvonne Drewes, Leiden University Medical Center
Comments to the Author:
Interesting manuscript, well written and very well structured.
Response: Thank you for your positive comments and we truly appreciate it.

Only some smaller points:
Regarding the abstract: The last sentence, 'Interventions to address VI might be a viable approach to improve cognitive function and the quality of life and well-being of elderly residents in residential care' is too far from the results, as this is a cross-sectional design; causal conclusions cannot be formulated based on these results
Response: Thank you. We agree with the comment. We have modified the last sentence in our revision as follows

“Interventions can be planned to address VI in this vulnerable population.”

Regarding the methods: were participants allowed to wear their spectacles during the vision test?
Response: Yes, the visual acuity was assessed with spectacles, if they reported using them. We have revised the text to clarify this.
Regarding the limitation section: I was wondering what are the indications to be admitted to a nursing home in India. Is a combination of impairments are needed? This could strongly influence the findings of this specific population. It is already mentioned that the results cannot be generalized to the population at large, but the authors can be more explicit about the entry requirements for a nursing home and the way this influenced the outcomes of this study.
Response: Thank you for this valuable comment. We have now included a short paragraph to provide clarity on homes for aged centres for the international audience and to provide context for the study.
“In India, homes for the aged are a recent and emerging concept. These homes are diverse in terms of scope, services provided and the number of individuals residing in the homes. There are no standardised guidelines for admission into these homes. While most homes admit people aged 60 and older, few even admit those aged 50 years and older. Few homes restrict admissions to older people with independent mobility while others also include those who need assistance for mobility and a few who are bedridden. In addition, there are specific homes which are typical nursing homes or rehabilitation centres for elderly with disabilities and those who are bedridden. Non-governmental, religious or voluntary organizations typically run these homes with support from the government and philanthropists. In some homes, either the elderly themselves or their kin pay the monthly or annual ‘user fee’.”

Reviewer: 2
Dr. Sumi Lee, Howon University

Comments to the Author:
Dear Srinivas Marmamula

I hope this message finds you well. I want to express our gratitude for submitting your manuscript entitled "Cognitive impairment and vision impairment among the elderly population in residential care in India: The Hyderabad Ocular Morbidity in Elderly Study (HOMES)]" to the BMJ reviewer. We appreciate the effort and dedication you have invested in your research.
Response: Thank you for your positive comments and we truly appreciate it.
After careful consideration by our reviewer team, it is with regret that we are unable to accept your manuscript for publication at this time. Our decision is based on a comprehensive evaluation, and we would like to provide you with specific feedback to help you enhance the quality of your work for future submissions.
Response: Thank you for your comments. We have addressed all the comments in our revision.

Abstract
In one sentence, unlike the other sentences, the p value is missing and needs to be corrected.
The logistic regression analysis showed that the participants with VI for distance vision were three times more likely to have CI (OR: 3.09; 95% Confidence Intervals: 2.13 – 4.47;<0.01).
Response: Thank you for pointing this out. We have corrected this in our revision.

Methods
I hope that the explanation of Near VI in Eye Health Assessment will be revised to be more concise and clear. In particular, it is difficult to understand what N8 means.

Response: We have added text in the methods section to more details on distance and near vision assessment. N8 corresponds to the standard font size used in the newsprint. The following text is added in our revision.
“”
“Visual acuity was assessed with spectacles, if available. Distance visual acuity was assessed at three meters in a well-illuminated room (at least 180 lux) and near vision was assessed at a fixed distance of 40 cm. Visual acuity charts with tumbling E optotypes and English letter alphabets were used for assessment.”

References
47 references are presented in the text. Among the references, it is recommended that numbers 2, 5, 34, 35, 36, and 41 be replaced with the most recent literature or deleted if possible.
Response: Thank you. We have updated the references with the more recent ones wherever available. A couple of them are seminal works that are relevant to our study hence we have retained them. I appreciate your consideration.

We encourage you to address these issues in a revised version of your manuscript before resubmitting it for consideration. We appreciate your understanding and hope that this feedback proves valuable in refining your work. If you have any questions or need further clarification, please feel free to contact us.
Response: Thank you for all the thoughtful comments to improve our manuscript. Truly appreciate the effort and time that have gone into the review of our manuscript. We have addressed all the comments and suggestions made.

Thank you for considering BMJ Open as a venue for your research, and we wish you success in your future scholarly endeavors.
Response: Thank you!

VERSION 2 – REVIEW

**REVIEWER**	Drewes, Yvonne<br>Leiden University Medical Center, Public Health and Primary Care
**REVIEW RETURNED**	08-May-2024

**GENERAL COMMENTS**	The remarks of the first review are well adressed.
